# Elementary Chemistry, Theoretical and Practical

**Published:** 1854-04

**Authors:** 


For sale by MILLER, ORTON & MULLIGAN. H.

---

ART. VII.—*Elementary Chemistry, Theoretical and Practical.* By GEORGE FOWNES, F. R. S., late Professor of Practical Chemistry in University College, London. Edited, with additions, by ROBERT BRIDGES, M. D. A new American, from the last and revised London edition. Philadelphia: Blanchard & Lea.

This well-known text-book for students is not intended as a complete manual of chemistry, but rather as an introduction to those other, and more advanced works, which have been published by other hands.

The numerous editions through which it has passed, indicate the estimation in which it is held, and endorse its value.

Of course it is not to be expected that we should enter upon a critical examination of a book so widely known. We can only chronicle the fact of a new edition, and inform our readers that the paper is good, and the type clear. It has another, and a very important, merit in a work on chemistry. Its wood-cuts are well engraved, and well subserve the purposes of illustration.

